# Eucalyptal D Enhances the Antifungal Effect of Fluconazole on Fluconazole-Resistant *Candida albicans* by Competitively Inhibiting Efflux Pump

**DOI:** 10.3389/fcimb.2019.00211

**Published:** 2019-06-20

**Authors:** Jiali Xu, Ruihuan Liu, Fujuan Sun, Lin An, Zhichun Shang, Lingyi Kong, Minghua Yang

**Affiliations:** State Key Laboratory of Natural Medicines, Department of Natural Medicinal Chemistry, China Pharmaceutical University, Nanjing, China

**Keywords:** fluconazole resistance, *Candida albicans*, eucalyptal D, efflux pump substrate, synergism, formyl-phloroglucinol meroterpenoids

## Abstract

The frequent emergence of azole-resistant strains has increasingly led azoles to fail in treating candidiasis. Combination with other drugs is a good option to effectively reduce or retard its incidence of resistance. Natural products are a promising synergist source to assist azoles in treating resistant candidiasis. Eucalyptal D (ED), a formyl-phloroglucinol meroterpenoid, is one of the natural synergists, which could significantly enhance the anticandidal activity of fluconazole (FLC) in treating FLC resistant *C. albicans*. The checkerboard microdilution assay showed their synergistic effect. The agar disk diffusion test illustrated the key role of ED in synergy. The rhodamine 6G (R6G) efflux assay reflected ED could reduce drug efflux, but quantitative reverse transcription PCR analysis revealed the upregulation of *CDR1* and *CDR2* genes in ED treating group. Efflux pump-deficient strains were hyper-susceptible to ED, thus ED was speculated to be the substrate of efflux pump Cdr1p and Cdr2p to competitively inhibit the excretion of FLC or R6G, which mainly contributed to its synergistic effect.

## Introduction

Candidiasis is the most frequently encountered fungal infection. Recent years have seen its steadily increasing incidence and mortality with risk factors continually emerging (Gamarra et al., 2010; Ruhnke, 2014; Tsai et al., 2015; Li et al., 2016). The azoles such as fluconazole (FLC), itraconazole (ICZ) and ketoconazole (KCZ) are widely and frequently used in clinic to fight candidiasis due to their high efficiency and low toxicity (Marchetti et al., 2003; Prasad and Rawal, 2014; Shrestha et al., 2015; Lu et al., 2017). However, it should be of concern that the continuing emergence of azole-resistant candidiasis leads to frequent failures in clinical treatment (Ahmad et al., 2013; Eddouzi et al., 2013; Liu et al., 2014; Shrestha et al., 2017).

The reason for azoles resistance could be multi-aspect. One of them is the increase of membrane transporters, such as Cdr1p, Cdr2p, and Mdr1p, which can significantly increase drug efflux to allow tolerable intracellular drug concentration for *Candida* spp. Another important factor is the overexpression or point mutation of *ERG11* gene, which can alter the affinity of azoles with target enzyme lanosterol 14-demethylase (Luna-Tapia et al., 2015). Clearly, the current new drugs discovery and development is not efficient enough to combat the rising azole-resistant problems (Harvey et al., 2015). Combination therapy might be an effective way to treat severe fungal infections and to reduce or retard the inducing incidence of resistant strains (Ghannoum and Elewski, 1999; Pinavaz et al., 2005; Holmes et al., 2016). Thus, the combined use of drugs or adjuncts with azoles is now increasingly popular in academic research for the treatment of azole-resistant candidiasis.

**Figure 1:**
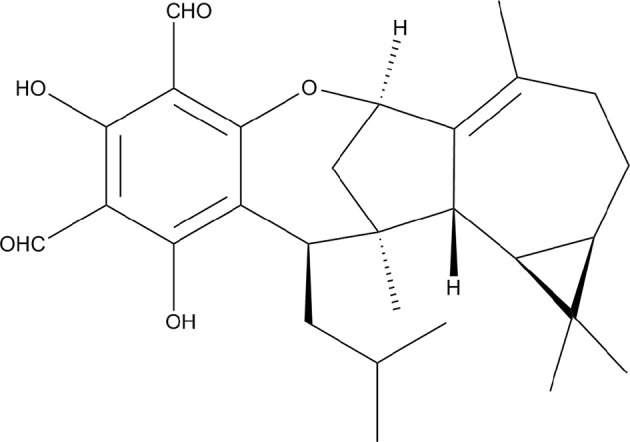
The chemical structure of ED.

The structural variety of natural products makes them a great resource for azole synergists, some of which were already found to be promising. For instance, the fungal metabolized immunosuppressive agents, cyclosporine A (CsA) and tacrolimus (FK506) were found to have synergistic effect with FLC in treating FLC resistant strains (Marchetti et al., 2000; Sun et al., 2008; Uppuluri et al., 2008; Cui et al., 2015). Several other natural products such as diorcinol D, osthole, and garlic also showed significant synergistic activity with FLC, although they were all weak alone (Li et al., 2015a, 2016; Li D. D. et al., 2017). In addition, Formyl-phloroglucinol meroterpenoids (FPMs), which are unique secondary metabolites of *Eucalyptus* and *Psidium* genera, have been recognized for their antifungal activities against pathomycetes like *Candida* and *Trichophyton spp* (Musyimi and Ogur, 2008; Wong et al., 2015). Our previous research also demonstrated that several novel FPMs exerted antifungal and antibiofilm activities against *Candida* spp. (Liu R. H. et al., 2017). Further investigations on FPMs showed that those FPMs with weak or without antifungal effects demonstrated enhanced activities when combined with other antifungal FPMs or azoles (data not shown). In this study, eucalyptal D (ED, Figure 1), an FPM, was revealed to have a strong efficacy when in synergy with azoles to reverse the resistance of *C. albicans in vitro*. However, ED upregulated the expression of efflux pump genes and could not synergistically inhibit *CDR*-deficient strains with azoles, which along with other experiments, indicated that ED functioned as a more competitive substrate of the Cdr1p and Cdr2p than azoles, and thus inhibited the excretion of azoles.

## Materials and Methods

### Strains

One reference strain (SC5314), seven clinical isolates including five FLC resistant isolates (24D, 28I, CA102, CA901, and CA112869) and two FLC susceptible strains (CA13, CA21), four transporter-deletion mutant strains (DSY448, DSY653, DSY465, and DSY659) and their parent strain CAF2-1, were used in our study. *C. albicans* SC5314 was purchased from ATCC, USA. The transporter-deletion mutant strains, and clinical strains 24D, 28I were kindly provided by Prof. Hongxiang Lou from the department of Natural Product Chemistry in Shandong University. The clinical strains CA13, CA21, CA102, CA901, and CA112869 were kindly offered by Prof. Yuanying Jiang from the School of Pharmacy, Second Military Medical University. Strains employed in this study are listed in Table 1. All strains were routinely stored at −80°C in yeast-peptone-dextrose medium (YPD; 1% yeast extract, 2% peptone, and 2% dextrose), supplemented with 20% glycerol (vol/vol) and were subcultured on YPD plates twice at 35°C before each experiment.

**Table 1:** Strains used in this study.

**Strains**	**Remarks or genotype**	**MIC_50_**^c^** (μg/ml)**	**Source or references**
SC5314		0.25	Fonzi and Irwin, 1993; Li et al., 2015b
3 resistant strains*^a^*	Clinical isolates, fluconazole resistant	>128	Liu R. H. et al., 2017
2 susceptible strains*^b^*	Clinical isolates, fluconazole susceptible	0.25	Liu R. H. et al., 2017
CAF2-1	*ura3Δ::imm434/URA3*	0.25	Sanglard et al., 1997; Sanglard and Coste, 2015
DSY448	*cdr1Δ::hisG-URA3-hisG/cdr1Δ::hisG*	<0.06	Sanglard et al., 1997; Sanglard and Coste, 2015
DSY653	*cdr2Δ::hisG-URA3-hisG/cdr2Δ::hisG*	0.125	Sanglard et al., 1997; Sanglard and Coste, 2015
DSY465	*mdr1Δ::hisG-URA3-hisG/mdr1Δ::hisG*	0.25	Sanglard et al., 1996; Sanglard and Coste, 2015
DSY659	*cdr1Δ::hisG/cdr1Δ::hisG cdr2Δ::hisG-URA3-hisG/cdr2Δ::hisG*	None	Li et al., 2015a

a *FLC resistant strains, 24D, 28I, CA102, CA901, CA112869*.

b *FLC susceptible strains, CA13, CA21*.

c *MIC values of FLC against strains in the literature*.

### Chemicals

Fluconazole (Gibco, USA), ketoconazole (TargetMol, USA), and itraconazole (Gibco, USA) were obtained commercially. ED was previously isolated from the leaves of *E. robusta* in our laboratory, and its purity (>99.80%) was analyzed by high performance liquid chromatography (HPLC). ED was prepared in dimethyl sulfoxide to achieve stock solutions of 12,800 μg/ml, and FLC was prepared in sterile distilled water to a concentration of 5,120 μg/ml. Ketoconazole and itraconazole were dissolved in dimethyl sulfoxide to form stock solutions of 3,000 and 1,000 μg/ml, respectively. These stock solutions were all stored at −20°C.

### Antifungal Susceptibility Test

The minimum inhibitory concentrations (MIC) of ED and FLC against *C. albicans* strains were determined by the broth microdilution method based in the Clinical and Laboratory Standards Institute (CLSI) standard M27-A3 (CLSI, 2008). One hundred microliter serially diluted drug and 100 μl cells suspension with a final concentration of 0.5–2.5 × 10^3^ cells/ml were added into 96-well plates, then the plates were incubated at 35°C for 24 h. Optical densities at 540 nm (OD540) were measured by microplate reader (Tecan SUNRISE) and the MIC was defined as the concentration of drugs that inhibited 80% of cell growth. For the broth microdilution checkerboard assays, each drug was serially diluted 2-fold in RPMI 1640 as previously described (Tabbene et al., 2015). Briefly, the final ED concentrations ranged from 1 to 64 μg/ml and the final FLC concentrations ranged from 1 to 32 μg/ml for resistant isolates and from 0.0625 to 4 μg/ml for susceptible isolates. A 50-μl aliquot of each ED dilution and FLC dilution was added to the wells in the 2nd to 9th columns and the B to H lines, respectively. Row A and column 1 contained the ED and FLC alone respectively, and the well at the intersection of row A and column 1 was the drug-free one that served as the growth control. The 12th columns were performed in 200 μl RPMI1640 to act as negative controls. One hundred microliter aliquots cells to a final concentration of 0.5–2.5 × 10^3^ cells/ml were added to each well mentioned above. All the wells on the plate were filled with RPMI 1640 to a final volume of 200 μl. The plate was incubated for 24 h at 35°C. Drug interactions were analyzed using the following two different models: the fractional inhibitory concentration index (FICI) model and the percentage of growth difference (ΔE) model (Meletiadis et al., 2003; Sun et al., 2017). FICI model expressed as FICI = FIC of A + FIC of B = MIC_A_ comb /MIC_A_ alone + MIC_B_ comb /MIC_B_ alone, where MIC_A_ alone and MIC_B_ alone are the MICs of drugs A and B acting alone and MIC_A_ comb and MIC_B_ comb are the concentrations of drugs A and B at the effective combinations, respectively. The FICI was interpreted as synergy when it was ≤ 0.5, as antagonism when it was >4.0, and any value in between was considered indifferent (Li D. D. et al., 2017). ΔE model was calculated as ΔE = E _predicted_-E _measured_, where E _predicted_ and E _measured_ are the predicted and measured percentages of growth with drugs A and B at diverse concentrations, respectively. When the ΔE value was positive, synergy was concluded, and higher ΔE values suggested stronger synergistic interactions. Conversely, negative ΔE values represent antagonism (Meletiadis et al., 2003). The ΔE values of each combination were used to depict three-dimensional plot and contour plot by OriginPro 7.5.

### Agar Disk Diffusion Test

Disk diffusion testing was done by following the previously described method with a few modifications (Quan et al., 2006; Li et al., 2015b). The *C. albicans* 24D was used in this test. Briefly, 3 ml of aliquot of 5 × 10^5^ cells/ml suspension was spread uniformly onto a series of yeast peptone dextrose (YPD) agar plates containing ED in different concentrations. A plate with 1% DMSO was used as the vehicle control. The 6-mm-diameter filter disks impregnated with FLC were placed onto the agar surface. The growth inhibition zones were measured after the plates were incubated at 35°C for 24 h. Images were collected using ChemiDOC^TM^ XRS + system with Image Lab™ software.

### Quantitative Reverse Transcription PCR

Quantitative reverse transcription PCR (qRT-PCR) was performed to detect the expression levels of efflux pump-related genes (*CDR1, CDR2*, and *MDR1*) affected by ED. *C. albicans* 24D cells were grown overnight in YPD medium and diluted to a cell density of 1.0 × 10^6^ cells/ml in RPMI 1640 medium with treatment at final concentrations of FLC 2 and ED 32 μg/ml. Cultures without drugs served as the control. After the incubation at 35°C for 12 h, cells were harvested for RNA extraction. Total RNAs were isolated using a Yeast RNAiso kit (Takara Bio, China), and OD_260/280_ was between 1.8-2.2. cDNA was synthesized using the kit HiScript II Q RT SuperMix for qPCR (+gDNA wiper) (Vazyme, Biotech) and diluted five times. qRT-PCR was mixed with cDNA (2 μl), and then fast started with SYBR Green Master Mix kit (Vazyme, Biotech) and gene primers in a final volume of 20 μl. The *ACT1* gene was used as the endogenous control. qRT-PCR was carried out on the QuantStudio^®^ 3 real-time PCR System (Applied Biosystems). Primers used in this study referred to Yi et al. (2017) and were listed in Supplementary Table 1. The relative expression levels of target genes were calculated by the 2^−ΔΔ*CT*^ method (Livak and Schmittgen, 2001; Rybak et al., 2017). All experiments were performed three times independently from each other.

### R6G EffluxAssay

Rhodamine 6G (R6G) efflux was assessed to determine whether transport proteins activity could be affected by ED as a previously described protocol (Liu X. et al., 2017). Briefly, single colonies of *C. albicans* 24D were inoculated into liquid YPD medium and grown overnight at 35°C. The cells were resuspended in glucose-free PBS (pH = 7.0) to a final concentration of 1 × 10^8^ cells/ml to de-energized for 2 h after being centrifuged and washed three times. Then, a final R6G concentration of 10 μM was first added to the above-mentioned cells and incubated at 35°C for 55 min. After the R6G uptake into cells was terminated with an ice-water bath for 10 min, the cells were harvested and washed three times with glucose-free PBS to remove the extracellular R6G, and ED was added to the suspension at the concentration of 32 μg/ml. A drug-free sample with only R6G was used as the control group. Then, each 100 μl sample of the extracellular remaining R6G was detected at specific time intervals after the addition of 0.1 M glucose (5, 10, 20, 30, 60 min) and centrifuged at 12,000 g for 30 s. The fluorescence of the supernatant was recorded with Thermo Scientific Varioskan Flash using the SkanIt software (excitation and emission wavelengths of 529 nm and an emission wavelength of 553 nm). All experiments were performed three times.

### Spot Assay

Spot assays were performed to measure susceptibilities of transporter-deletion mutant strains to ED and FLC as described elsewhere (Kumar et al., 2014). Briefly, cells were grown overnight in YPD medium at 30°C and adjusted to an initial cell density of 1.0 × 10^6^ cells/ml and were 5-fold serially diluted. Then 5 μl aliquots of serial dilution cells were spotted onto YPD medium containing 128 μg/ml ED. Cultures without drugs served as the controls. The differences in plates after incubation at 30°C for 48 h were visualized and pictures were taken with ChemiDOCTM XRS + system with Image Lab™ software.

### Cell Toxicity Assay

The MTT [3(4,5-dimethylthiazol-2yl) 2,5-diphenyltetrazolium bromide] method was used to evaluate its cytotoxicity *in vitro* Jeon et al., 2014; Liu et al., 2016. Briefly, the LO2 (normal human hepatic cell line) and MCF 10A (normal breast cell line) cell lines were seeded into 96-well plates (5 × 10^3^ cells/well) and then incubated overnight, followed by treatment with various concentrations of ED. After 48 h for LO2 and 24 h for MCF 10A, respectively, the MTT solution was added to each well. After further incubation for 4 h, DMSO was added to dissolve the dark formazan crystals. Optical densities at 570 nm (OD570) were measured by microplate reader (Tecan SUNRISE).

### Statistical Analysis

Group data are presented as mean ± SD. Two-way RM ANOVA was used to determine differences between means. Differences were considered significant at *P* < 0.05. The GraphPad Prism (version 5.0) statistical package was used.

## Results

### Susceptibilities and Interactions of Drugs Against *C. albicans*

**Figure 2:**
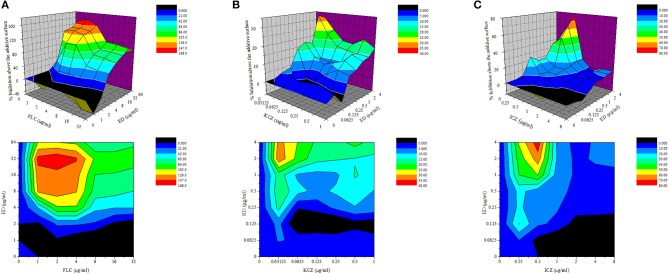
The drug interactions of ED and FLC **(A)**, KCZ **(B)**, and ICZ **(C)** against resistant *C. albicans* 24D are interpreted by the ΔE model. The 3D and contour plot were constructed by using OriginPro 7.5. The concentrations of FLC and ED are depicted on the x axis and y axis, respectively, and the ΔE values obtained for each combination is depicted on the z axis. Positive ΔE values represent synergy, and higher ΔE values suggested stronger synergistic interactions. Negative ΔE values represent antagonism.

Five FLC resistant strains, two FLC susceptible isolates and a reference strain were used to evaluate the anti-*Candida* actions of ED and its combination with FLC. Although ED was active against FLC susceptible strains with MIC value lower than 64 μg/ml, it didn't exhibit evident effects against FLC resistant isolates under the concentration of 128 μg/ml. However, when ED was simultaneously incubated with FLC, the combination showed strong synergistic effects (Table 2). With the help of ED, the concentrations of FLC exerted the same MIC_80_ values decreased 8-fold and more than 64-fold when treating against susceptible and resistant strains, respectively. The FICI values below 0.5 revealed the synergy between two drugs. The three-dimensional (3D) and contour plot constructed with ΔE values further demonstrated the synergy of most combinations, while the treatment with concentration lower than 2 μg/ml of ED showed almost all peaks below the zero planes, indicating their antagonistic effects (Figure 2A). The succedent evaluation of ED with KCZ and ITZ, respectively, resulted in a similar synergetic effect. ED remarkably reduced their doses in killing all five tested FLC resistant isolates with 4-fold to more than 256-fold potentiation effects (Table 3). Similarly, the 3D plot and contour plot also confirmed their synergies and showed antagonism at the low concentration of ED (Figures 2B,C). It was clear that ED could significantly improve the effect of azoles, especially on FLC resistant strains.

**Table 2:** *In vitro* susceptibilities of ED and FLC alone and in combination against *C. albicans*.

**Strains**	**MIC_80_ of drugs*^a^* (μg/ml)**				**FICI*^b^***
	**Alone**		**Combination**		
	**ED/FLC**		**ED/FLC**		
24D*^c^*	>128	>128	32	2	0.266
28I*^c^*	>128	>128	2	2	0.031
CA102*^c^*	>128	>128	4	8	0.094
CA901*^c^*	>128	>128	4	2	0.047
CA112869*^c^*	>128	>128	4	4	0.063
CA13*^d^*	64	2	2	0.25	0.156
CA21*^d^*	64	2	2	0.25	0.156
SC5314*^d^*	32	2	4	0.25	0.250

a *ED, eucalyptal D; FLC, fluconazole*.

b *Synergism and antagonism were defined by FICI of ≤ 0.5 and >4, respectively. An FICI index result of >0.5 but ≤ 4 was considered indifferent*.

c *FLC resistant strains*.

d *FLC susceptible strains*.

**Table 3:** Interactions of other azoles and ED against five FLC resistant isolates by microdilution assay.

**Clinical isolates**	**MIC_80_ (μg/ml) alone*^a^***			**MIC_80_ (μg/ml) in combination**		**FICI*^b^***	
	**KCZ**	**ICZ**	**ED**	**KCZ/ED**	**ICZ/ED**	**KCZ/ED**	**ICZ/ED**
24D	1	>8	>128	0.03125/2	2/0.5	0.047	0.253
28I	16	>8	>128	0.125/8	2/8	0.070	0.312
CA102	4	>8	>128	0.25/4	0.5/8	0.093	0.128
CA901	>16	>8	>128	0.0625/4	0.5/8	0.035	0.128
CA112869	16	>8	>128	0.0625/8	1/8	0.066	0.187

a *ED, eucalyptal D; KCZ, ketoconazole; ICZ, itraconazole*.

b *Synergism and antagonism were defined by FICI of ≤ 0.5 and >4, respectively. An FICI index result of >0.5 but ≤ 4 was considered indifferent*.

### Interactions of ED and FLC by Agar Disk Diffusion Test

**Figure 3:**
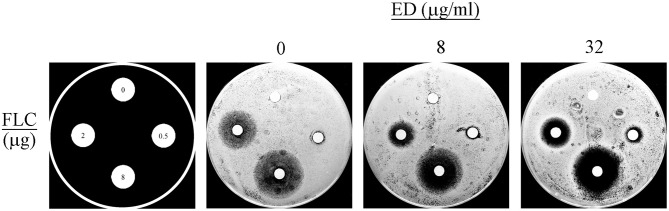
The disk diffusion assay results. The assays were performed by plating *albicans* 24D (5 × 10^5^ cells/ml) on the agar plates in the absence (DMSO vehicle alone) or presence of ED at the concentration of 8 and 32 μg/ml. FLC was used at 0, 0.5, 2, and 8 μg per disc, respectively. Each plate was incubated at 35°C for 24 h. Large and clear inhibition zones represent better antifungal effect.

The agar diffusion assay allowed an intuitionistic observation of the combination effects. A dozen combinations with various concentrations of ED and FLC were applied as shown in Figure 3. When using FLC alone, all the three concentration groups did not show any distinct inhibition zone. By contrast, FLC in different concentrations yielded positive inhibition zones with the help of ED, even when the FLC concentration was a quarter of the optimal combination concentration (0.5 μg/ml). And the inhibition zones produced by the same quality of FLC were more distinct and clearer when concentrations of ED increased. Thus, there was no doubt about the important role of ED in synergistic antifungal action.

### Effect of ED on the Expression Levels of Efflux Pump and Other Genes

**Figure 4:**
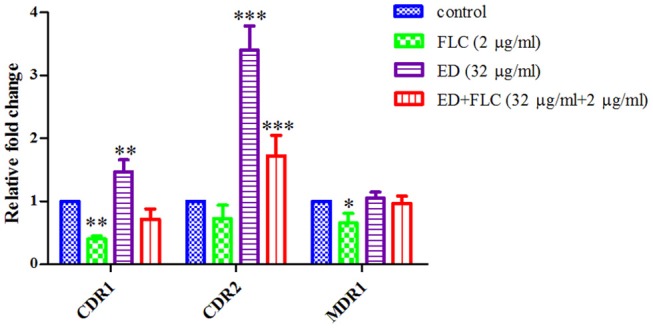
The relative expression levels of efflux genes *CDR1, CDR2*, and *MDR1* in FLC resistant strain 24D. Cells were treated with FLC (2 μg/ml), ED (32 μg/ml), and their combination, respectively. Data are normalized for control group. *ACT1* was used as an expression control. Relative fold change above one represents gene upregulation, while below one represents gene downregulation. Data are means ± SD from three experiments. ^*^p < 0.05, ^**^p < 0.01, ^***^p < 0.001.

The expression levels of efflux pump genes were quantified by qRT-PCR, along with those of ergosterol biosynthesis genes related to FLC resistance. Interestingly, the results showed that ED alone could upregulate expression level of *CDR1* and *CDR2*, while the expression level of *MDR1* remained nearly unchanged. The expression level of *CDR2* was the most upregulated, which was 3.40 (alone) and 1.72 (with FLC) times higher than that of the control (Figure 4). All the three gene levels of combination groups were the compromise of ED and FLC effect (see details in Supplementary Table 2). In the meantime, the relative expression levels of ergosterol biosynthesis genes were evaluated to see if there was any other synergetic pathway (Supplementary Figure 4). Although the combination led to the overexpression of several ergosterol biosynthesis genes, these results were mostly due to FLC which alone could also induce similar expression levels. Thus, ED mainly affected *CDR1* and *CDR2* expressions in tested strains.

### Effect of ED on Inhibiting Drug Efflux of Resistant *C. albicans*

**Figure 5:**
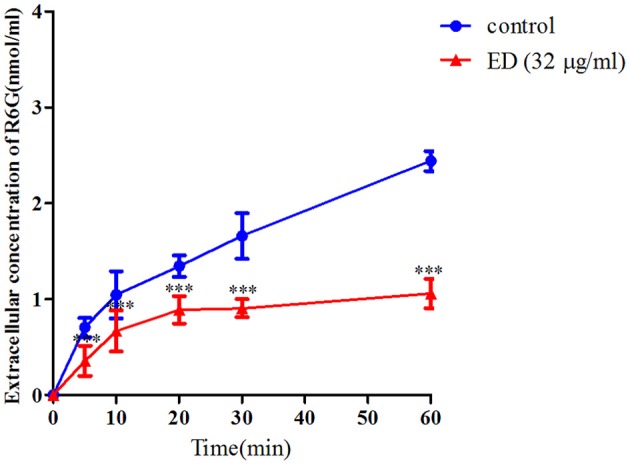
The effect of ED on the efflux of R6G in FLC resistant strain 24D. The R6G fluorescence of supernatant in the absence and presence of ED were determined with a Varioskan Flash (Thermo Scientific) using the SkanIt software. The extracellular concentration of R6G was calculated by using the R6G standard curve. Data are means ± SD from three experiments, ^***^p < 0.001, compared with the control.

The R6G efflux assay was further used to verify whether ED could influence drug efflux. As shown in Figure 5, the extracellular concentration of R6G in two groups steadily increased at first, while the graph of ED treating group flattened out gradually after 20 min incubation. After 60 min, the R6G concentration of ED group (1.05 ± 0.15 nmol/ml) was about 43.0% of the control (2.44 ± 0.10 nmol/ml). These data obviously revealed that ED could inhibit the efflux of intracellular R6G, and that the predictable same inhibitory effect on azoles efflux would be responsible for the interaction between ED and azoles (see details in Supplementary Table 3).

### Susceptibilities of ED Against Transporter-Deletion Mutants

**Figure 6:**
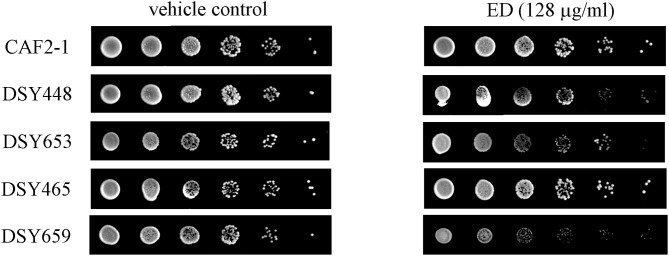
The susceptibility of ED and FLC against transporter-deletion mutants by spot assay. Cells were adjusted to 1 × 10^7^ cells/ml, 5-fold serially diluted, and spotted onto YPD medium containing 128 μg/ml ED or 1% DMSO (vehicle control). The plates were incubated at 30°C for 48 h. less colony means more sensitive to drug.

The upregulation of pump genes seemed inconsistent with the result of R6G efflux assay, since the overproduction of efflux pumps caused by their upregulated genes should have afforded a higher extracellular concentration of R6G. To further verify the results of R6G efflux assay, susceptibility test, and spot assay were carried out on transporter-deletion mutants. In the drug susceptibility test (Table 4), MIC_80_ values of ED in *CDR1* deficient strain were reduced by more than 50% (from more than 128 μg/ml to lower than 64 μg/ml). Although no activity change was found in the *CDR*2 deficient strain, inhibition in double mutant strain DSY659 was two times stronger when compared with that of *CDR1* deficient strain. Susceptibility was unchanged in *MDR1* deficient strain. Similar results were revealed in spot assay. The ABC transporter-deletion mutants DSY448 (*cdr1*Δ*/*Δ), DSY653 (*cdr2*Δ*/*Δ), and DSY659 (*cdr1*Δ*/cdr2*Δ) were hypersensitive to ED. The strain DSY659 (*cdr1*Δ*/cdr2*Δ) was especially more sensitive to ED than the parent strain CAF2-1 (Figure 6). More significantly, ED failed to enhance FLC activity when the combinations were facing the double mutant DSY659 (*cdr1*Δ*/cdr2*Δ) in checkerboard microdilution assay (Supplementary Figure 1).

**Table 4:** In *vitro* susceptibilities of ED against transporter-deletion mutants.

**Strains**	**MIC_80_ of drugs*^a^* (μg/ml)**	
	**ED**	**FLC**
CAF2-1	>128	0.5
DSY448(*cdr1Δ/Δ*)	64	0.0625
DSY653(*cdr2Δ/Δ*)	>128	0.125
DSY465(*mdr1Δ/Δ*)	>128	0.5
DSY659(*cdr1Δ/cdr2Δ*)	32	0.0625

a *ED, eucalyptal D; FLC, fluconazole*.

### Cytotoxicity of ED on Human Normal Cell Lines

The *in vitro* cytotoxic effect was tested on LO2 and MCF 10A cell lines. As showed in Supplementary Figure 2, ED at the concentration of 32 μg/ml had a slight inhibition on LO2 cell lines and no cytotoxicity on MCF 10A cell lines.

## Discussion

Drug combination is commonly used to treat various illnesses. It helps to increase drug potency and to decrease toxicity, meanwhile it allows to retard the potential drug resistance and to recover the potency of resistant drugs (Cui et al., 2015; Li et al., 2015a). Drug combination is also a valid and pragmatic strategy to overcome resistance in *C. albicans* infections (Gu et al., 2016; Sun et al., 2016). There were many antifungal researches on drug combination, which investigated not only clinical drugs but also their combination with natural products. Some non-antifungal agents such as Hsp90 inhibitor geldanamycin and calcium homeostasis regulator amiodarone were found to be useful in improving activities of antifungal drugs (Gamarra et al., 2010; Hill et al., 2013; Liu et al., 2014). Some natural products did display prominent synergistic effects when used in combination with azoles (Li et al., 2016; Li D. D. et al., 2017; Sun et al., 2017). Yet to our knowledge, most of the combinations were to increase the intercellular concentration by reducing the overexpressed efflux pump genes (Eddouzi et al., 2013; Sarah et al., 2016).

Although ED showed weak antifungal activity, it was found to exert outstanding synergistic effect with FLC, KCZ, and ICZ on FLC resistant *C. albicans* strains (Tables 2, 3). Reconfirmation was conducted by the non-parametric ΔE model based on BI theory (Meletiadis et al., 2003; Sun et al., 2008), which verified the synergy effect of ED with azoles at different concentrations. Meanwhile, it was interesting to find that ED below specific low concentrations yielded antagonistic effects in all the combinations with tested azoles (Figures 2A–C). The antagonism was rarely observed in previous combination studies using dexamethasone, thymol, and carvacrol which were able to downregulate the expression of drug efflux pump genes (Ahmad et al., 2013; Sun et al., 2017). Thus, a different action of ED could be responsible for its synergetic effect. Subsequently, we tried to figure out the role played by ED in the synergy effect. As was shown by its inhibition zones in the agar disk diffusion test, the inhibition of FLC against *C. albicans* was dose dependent. However, complete inhibition could only be achieved by the participation of ED (Figure 3). Obviously, ED could assist FLC in killing resistant *C. albicans* when applying them together.

By quantifying the gene expression level, we knew that the resistance of tested strain 24D was mainly due to the overexpression in genes of ABC, MFS (major facilitator superfamily) plasma membrane transporter, and *ERG11* key gene encoding FLC target enzyme lanosterol 14-demethylase (Supplementary Table 4). This clue gave priority to find out if there would be any differences on gene expression levels after treating with ED. It was unforeseen that ED led to the upregulation of *CDR1* and *CDR2* genes (Figure 4) which was always correlated with the increase of drug efflux, resulting in the resistance to FLC (Eddouzi et al., 2013; Bhattacharya et al., 2016; Sun et al., 2017). However, ED not only induced the overexpression of efflux pump genes but also simultaneously helped FLC to reverse the resistance. At the same time, it was a little unusual that FLC slightly downregulated the level of *CDR1* and *CDR2*. Investigations on references indicated that this downregulation could be due to the strain's peculiarity (Guo et al., 2014; Wei et al., 2016), the treatment concentration or time of FLC (De Backer et al., 2001; Lepak et al., 2006). An additional test was carried out on other drug-resistant and susceptible strains (28I and CA21). Both of them overexpressed the *CDR* genes after treatment with either FLC or ED (Supplementary Figure 5). Therefore, ED could consistently upregulate the expression of efflux pump genes in different *C. albicans* strains. The accumulation of fluorescent dyes at different time intervals showed the ability of efflux pump (Kolaczkowski et al., 2009). The dye R6G can be used to identify the *CDR* pump-mediated azole-resistant strains (Maesaki et al., 1999; Holmes et al., 2016), and R6G assay is also widely applied in evidencing whether synergists could reduce the extrusion of drugs (Ahmad et al., 2013; Sun et al., 2017). In our test, the R6G assay undoubtfully confirmed that ED could reduce the efflux of FLC. This inhibition obviously surpassed ED's simultaneous effect on the upregulation of efflux pump genes, otherwise ED could not reverse the resistance to FLC. Further literature investigations revealed that fluphenazine, a known substrate of Cdr1p and Cdr2p, also had similar implications (Henry et al., 1999). It could competitively inhibit the efflux of FLC at high concentrations, and meanwhile could upregulate the expression of efflux pump genes. Judging from the similar behavior, we thought that ED might have played the same role as a substrate of Cdr1p and Cdr2p. One of the strategies used to screen for substrates of efflux pumps is to test drugs on the mutants of efflux pump genes (Sanglard et al., 1996; Tsao et al., 2009; Szczepaniak et al., 2015; Li S. et al., 2017; Chang et al., 2018). A series of efflux pump deletion strains were thus used to confirm whether and which efflux transporters were affected by ED. Compared with the parental strain *C. albicans* CAF2-1, the *CDR1* deficient strain was more susceptible to ED (Table 4 and Figure 6), which meant that ED was pumped out mainly by Cdr1p in wild strains. Considering the synergistic effect with azoles, ED was most likely a more competitive substrate for Cdr1p than azoles. In line with this, ED could not assist FLC anymore when used against double mutant strain DSY659 (*cdr1*Δ*/cdr2*Δ) (Supplementary Figure 1). We tried to directly quantify the variation of extracellular FLC concentrations in vain due to the poor quantification limit of microspectrophotometer and HPLC detector. Nevertheless, the competitive inhibition on FLC efflux is the most likely mechanism for the synergistic effect of ED according to this study. In addition, since ED alone did show weak inhibition on susceptible strains, its inhibiting effect could be another synergistic factor when combined with azoles. Yet it's not the key factor as no synergy was found when the combination facing *CDR* double mutants.

## Conclusions

In this study, azoles were found to significantly restore their antifungal activities against azole-resistant *C. albicans* when they were applied in combination with ED, a formyl-phloroglucinol meroterpenoid. Differently from the other synergists that normally reduce the expression level of efflux pump genes, ED was found to competitively inhibit FLC from being excreted. It suggests that FPMs like ED are hopeful to be developed as an antifungal adjunct of azoles.

## Author Contributions

JX, FS, LA, and ZS performed the experiments. JX and RL designed the research. JX and MY analyzed the data and wrote the paper. LK reviewed the manuscript and the experiments. All authors read and approved the final manuscript.

### Conflict of Interest Statement

The authors declare that the research was conducted in the absence of any commercial or financial relationships that could be construed as a potential conflict of interest.

## References

[B1] Ahmad A. Khan A. Manzoor N. (2013). Reversal of efflux mediated antifungal resistance underlies synergistic activity of two monoterpenes with fluconazole. Eur. J. Pharm. Sci. 48, 80–86. 10.1016/j.ejps.2012.09.016 23111348

[B2] Bhattacharya S. Sobel J. D. White T. C. (2016). A combination fluorescence assay demonstrates increased efflux pump activity as a resistance mechanism in azole-resistant vaginal *Candida albicans* isolates. Antimicrob. Agents Chemother. 60, 5858–5866. 10.1128/AAC.01252-16 27431223 PMC5038269

[B3] Chang W. Liu J. Zhang M. Shi H. Zheng S. Jin X.. (2018). Efflux pump-mediated resistance to antifungal compounds can be prevented by conjugation with triphenylphosphonium cation. Nat. Commun. 9:5102. 10.1038/s41467-018-07633-9 30504815 PMC6269435

[B4] CLSI (2008). Reference Methods for Broth Dilution Antifungal susceptibility testing of yeast; Approved Standard-third edition, CLSI Document M27-A3. Wayne, PA: CLSI.

[B5] Cui J. Ren B. Tong Y. Dai H. Zhang L. (2015). Synergistic combinations of antifungals and anti-virulence agents to fight against *Candida albicans*. Virulence. 6, 362–371. 10.1080/21505594.2015.1039885 26048362 PMC4601232

[B6] De Backer M. D. Ilyina T. Ma X. J. Vandoninck S. Luyten W. H. Vanden B. H. (2001). Genomic profiling of the response of *Candida albicans* to itraconazole treatment using a dna microarray. Antimicrob. Agents Chemother. 45, 1660–1670. 10.1128/AAC.45.6.1660-1670.2001 11353609 PMC90529

[B7] Eddouzi J. Parker J. E. Valesilva L. A. Coste A. Ischer F. Kelly S.. (2013). Molecular mechanisms of drug resistance in clinical *Candida* species isolated from tunisian hospitals. Antimicrob. Agents Chemother. 57, 3182–3193. 10.1128/AAC.00555-13 23629718 PMC3697321

[B8] Fonzi W. A. Irwin M. Y. (1993). Isogenic strain construction and gene mapping in *Candida albicans*. Genetics. 134, 717–728. 8349105 10.1093/genetics/134.3.717 PMC1205510

[B9] Gamarra S. Rocha E. M. Zhang Y. Q. Park S. Rao R. Perlin D. S. (2010). Mechanism of the synergistic effect of amiodarone and fluconazole in *Candida albicans*. Antimicrob. Agents Chemother. 54, 1753–1761. 10.1128/AAC.01728-09 20194694 PMC2863688

[B10] Ghannoum M. A. Elewski B. (1999). Successful treatment of fluconazole-resistant oropharyngeal candidiasis by a combination of fluconazole and terbinafine. Clin. Diagn. Lab. Immunol. 6, 921–923. 10.1006/cimm.1999.1566 10548586 PMC95798

[B11] Gu W. Guo D. Zhang L. Xu D. Sun S. (2016). The synergistic effect of azoles and fluoxetine against resistant *Candida albicans* strains is attributed to attenuating fungal virulence. Antimicrob. Agents Chemother. 60, 6179–6188. 10.1128/AAC.03046-15 27503639 PMC5038273

[B12] Guo H. Xie S. M. Li S. X. Song Y. J. Lv X. L. Zhang H. (2014). Synergistic mechanism for tetrandrine on fluconazole against *Candida albicans* through the mitochondrial aerobic respiratory metabolism pathway. J. Med. Microbiol. 63, 988–996. 10.1099/jmm.0.073890-0 24790082

[B13] Harvey A. L. Edrada-Ebel R. A. Quinn R. J. (2015). The re-emergence of natural products for drug discovery in the genomics era. Nat. Rev. Drug. Discov. 14, 111–129. 10.1038/nrd4510 25614221

[B14] Henry K. W. Cruz M. C. Katiyar S. K. Edlind T. D. (1999). Antagonism of azole activity against *Candida albicans* following induction of multidrug resistance genes by selected antimicrobial agents. Antimicrob. Agents Chemother. 43, 1968–1974. 10.1128/AAC.43.8.1968 10428921 PMC89399

[B15] Hill J. A. Ammar R. Torti D. Nislow C. Cowen L. E. (2013). Genetic and genomic architecture of the evolution of resistance to antifungal drug combinations. PLoS Genet. 9:e1003390. 10.1371/journal.pgen.1003390 23593013 PMC3617151

[B16] Holmes A. R. Cardno T. S. Strouse J. J. Ivnitski-Steele I. Keniya M. V. Lackovic K.. (2016). Targeting efflux pumps to overcome antifungal drug resistance. Future. Med. Chem. 8, 1485–1501. 10.4155/fmc-2016-0050 27463566 PMC5827819

[B17] Jeon H. Kim J. H. Lee E. Jang Y. J. Lee K. W. (2014). Methionine deprivation suppresses triple-negative breast cancer metastasis *in vitro* and *in vivo*. Oncotarget. 7, 67223–67234. 10.18632/oncotarget.11615 27579534 PMC5341870

[B18] Kolaczkowski M. Kolaczkowska A. Motohashi N. Michalak K. (2009). New high-throughput screening assay to reveal similarities and differences in inhibitory sensitivities of multidrug ATP-binding cassette transporters. Antimicrob. Agents Chemother. 53, 1516–1527. 10.1128/AAC.00956-08 19188399 PMC2663063

[B19] Kumar A. Dhamgaye S. Maurya I. K. Singh A. Sharma M. Prasad R. (2014). Curcumin targets cell wall integrity via calcineurin-mediated signaling in *Candida albicans*. Antimicrob. Agents Chemother. 58, 167–175. 10.1128/AAC.01385-13 24145527 PMC3910804

[B20] Lepak A. Nett J. Lincoln L. Marchillo K. Andes D. (2006). Time course of microbiologic outcome and gene expression in *Candida albicans* during and following *in vitro* and *in vivo* exposure to fluconazole. Antimicrob Agents Chemother. 50, 1311–13119. 10.1128/AAC.50.4.1311-1319.2006 16569846 PMC1426956

[B21] Li D. D. Chai D. Huang X. W. Guan S. X. Du J. Zhang H. Y.. (2017). Potent *in vitro* synergism of fluconazole and osthole against fluconazole-resistant *Candida albicans*. Antimicrob. Agents Chemother. 61:e00436–17. 10.1128/AAC.00436-17 28607012 PMC5527582

[B22] Li S. Shi H. Chang W. Li Y. Zhang M. Qiao Y.. (2017). Eudesmane sesquiterpenes from chinese liverwort are substrates of *CDR*s and display antifungal activity by targeting *ERG6* and *ERG11* of *Candida albicans*. Bioorg Med Chem. 25, 5764–5771. 10.1016/j.bmc.2017.09.001 28935182

[B23] Li W. R. Shi Q. S. Dai H. Q. Liang Q. Xie X. B. Huang X. M.. (2016). Antifungal activity, kinetics and molecular mechanism of action of garlic oil against *Candida albicans*. Sci. Rep. 6:22805. 10.1038/srep22805 26948845 PMC4779998

[B24] Li Y. Chang W. Zhang M. Li X. Jiao Y. Lou H. (2015a). Synergistic and drug-resistant reversing effects of diorcinol d combined with fluconazole against *Candida albicans*. FEMS Yeast Res. 15:fov001. 10.1093/femsyr/fov001 25752309

[B25] Li Y. Wan Z. Liu W. Li R. (2015b). Synergistic activity of chloroquine with fluconazole against fluconazole-resistant isolates of *Candida* species. Antimicrob. Agents Chemother. 59, 1365–1369. 10.1128/AAC.04417-14 25512426 PMC4335839

[B26] Liu L. J. Wang W. Kang T. S. Liang J. X. Liu C. Kwong D. W. J.. (2016). Antagonizing stat5b dimerization with an osmium complex. Sci. Rep. 6:36044. 10.1038/srep36044 27853239 PMC5113070

[B27] Liu R. H. Shang Z. C. Li T. X. Yang M. H. Kong L. Y. (2017). *In vitro* antibiofilm activity of eucarobustol E against *Candida albicans*. Antimicrob. Agents Chemother. 61:e02707–16. 10.1128/AAC.02707-16 28584159 PMC5527566

[B28] Liu S. Hou Y. Chen X. Gao Y. Li H. Sun S. (2014). Combination of fluconazole with non-antifungal agents: a promising approach to cope with resistant *Candida albicans* infections and insight into new antifungal agent discovery. Int. J. Antimicrob. Agents. 43, 395–402. 10.1016/j.ijantimicag.2013.12.009 24503221

[B29] Liu X. Li T. Wang D. Yang Y. Sun W. Liu J.. (2017). Synergistic antifungal effect of fluconazole combined with licofelone against resistant *Candida albicans*. Front. Microbiol. 8:2101. 10.3389/fmicb.2017.02101 29163396 PMC5681995

[B30] Livak K. J. Schmittgen T. D. (2001). Analysis of relative gene expression data using real-time quantitative PCR and the 2(-delta delta c(t)) method. Methods.25, 402–408 10.1006/meth.2001.1262 11846609

[B31] Lu M. Yu C. Cui X. Shi J. Yuan L. Sun S. (2017). Gentamicin synergizes with azoles against resistant *Candida albicans*. Int. J. Antimicrob. Agents. 51, 107–114. 10.1016/j.ijantimicag.2017.09.012 28943366

[B32] Luna-Tapia A. Kerns M. E. Eberle K. E. Jursic B. S. Palmer G. E. (2015). Trafficking through the late endosome significantly impacts *Candida albicans* tolerance of the azole antifungals. Antimicrob. Agents Chemother. 59, 2410–2420. 10.1128/AAC.04239-14 25666149 PMC4356793

[B33] Maesaki S. Marichal P. Vanden B. H. Sanglard D. Kohno S. (1999). Rhodamine 6G efflux for the detection of *CDR1*-overexpressing azole-resistant *Candida albicans* strains. J. Antimicrob Chemother. 44, 27–31. 10.1093/jac/44.1.27 10459807

[B34] Marchetti O. Moreillon P. Glauser M. P. Bille J. Sanglard D. (2000). Potent synergism of the combination of fluconazole and cyclosporine in *Candida albicans*. Antimicrob. Agents Chemother. 44, 2373–81. 10.1128/AAC.44.9.2373-2381.2000 10952582 PMC90072

[B35] Marchetti O. Moreillon P. Josè M Entenza V. J. Glauser M. P. Bille J. (2003). Fungicidal synergism of fluconazole and cyclosporine in *Candida albicans* is not dependent on multidrug efflux transporters encoded by the *CDR1, CDR2, CaMDR1*, and *FLU1* genes. Antimicrob. Agents Chemother. 47, 1565–1570. 10.1128/AAC.47.5.1565-1570.2003 12709323 PMC153326

[B36] Meletiadis J. Mouton J. W. Meis J. F. G. M. Verweij P. E. (2003). *In vitro* drug interaction modeling of combinations of azoles with terbinafine against clinical scedosporium prolificans isolates. Antimicrob. Agents Chemother. 47, 106–117. 10.1128/AAC.47.1.106-117.2003 12499177 PMC149034

[B37] Musyimi D. Ogur J. (2008). Comparative assessment of antifungal activity of extracts from *eucalyptus globulus* and *eucalyptus citriodora*. Res. J. Phytochem. 2, 35–43. 10.3923/rjphyto.2008.35.43

[B38] Pinavaz C. Rodrigues A. Costadeoliveira S. Ricardo E. Mardh PerAnders. (2005). Potent synergic effect between ibuprofen and azoles on *Candida* resulting from blockade of efflux pumps as determined by fun-1 staining and flow cytometry. J. Antimicrob Chemother. 56, 678–685. 10.1093/jac/dki264 16115827

[B39] Prasad R. Rawal M. K. (2014). Efflux pump proteins in antifungal resistance. Front. Pharmacol. 5:202. 10.3389/fphar.2014.00202 25221515 PMC4148622

[B40] Quan H. Cao Y. Y. Xu Z. Zhao J. X. Gao P. H. Qin X. F.. (2006). Potent *in vitro* synergism of fluconazole and berberine chloride against clinical isolates of *Candida albicans* resistant to fluconazole. Antimicrob. Agents Chemother. 50, 1096–1099. 10.1128/AAC.50.3.1096-1099.2006 16495278 PMC1426442

[B41] Ruhnke M. (2014). Antifungal stewardship in invasive *Candida* infections. Clin. Microbiol. Infect. 20, 11–18. 10.1111/1469-0691.12622 24661820

[B42] Rybak J. M. Dickens C. M. Parker J. E. Caudle K. Manigaba K. Whaley S. G.. (2017). Loss of c-5 sterol desaturase activity results in increased resistance to azole and echinocandin antifungals in a clinical isolate of candida parapsilosis. Antimicrob Agents Chemother. 61:AAC.00651–17. 10.1128/AAC.00651-17 28630186 PMC5571332

[B43] Sanglard D. Coste A. T. (2015). Activity of isavuconazole and other azoles against candida clinical isolates and yeast model systems with known azole resistance mechanisms. Antimicrob Agents Chemother. 60:AAC.02157–15. 10.1128/AAC.02157-15 26482310 PMC4704203

[B44] Sanglard D. Ischer F. Monod M. Bille J. (1996). Susceptibilities of *Candida albicans* multidrug transporter mutants to various antifungal agents and other metabolic inhibitors. Antimicrob Agents Chemother. 40, 2300–2305. 10.1128/AAC.40.10.2300 8891134 PMC163524

[B45] Sanglard D. Ischer F. Monod M. Bille J. (1997). Cloning of *Candida albicans* genes conferring resistance to azole antifungal agents: characterization of *CDR2*, a new multidrug ABC transporter gene. Microbiology. 143, 405–416. 10.1099/00221287-143-2-405 9043118

[B46] Sarah T.S. Sandra W. Christine C. Dominic N. Elaheh A. Martine R. (2016). Positive regulation of the *Candida albicans* multidrug efflux pump Cdr1p function by phosphorylation of its n-terminal extension. J. Antimicrob. Chemother. 71, 3125–3134. 10.1093/jac/dkw252 27402010 PMC5079294

[B47] Shrestha S. K. Fosso M. Y. Garneautsodikova S. (2015). A combination approach to treating fungal infections. Sci. Rep. 5:17070. 10.1038/srep17070 26594050 PMC4655404

[B48] Shrestha S. K. Garzan A. Garneautsodikova S. (2017). Novel alkylated azoles as potent antifungals. Eur. J. Med. Chem. 133, 309–318. 10.1016/j.ejmech.2017.03.075 28395217

[B49] Sun S. Li Y. Guo Q. Shi C. Yu J. Ma L. (2008). *In vitro* interactions between tacrolimus and azoles against *Candida albicans* determined by different methods. Antimicrob. Agents Chemother. 52, 409–417. 10.1128/AAC.01070-07 18056277 PMC2224779

[B50] Sun W. Sanderson P. Zheng W. (2016). Drug combination therapy increases successful drug repositioning. Drug. Discov. Today. 21, 1189–1195. 10.1016/j.drudis.2016.05.015 27240777 PMC4907866

[B51] Sun W. Wang D. Yu C. Huang X. Li X. Sun S. (2017). Strong synergism of dexamethasone in combination with fluconazole against resistant *Candida albicans* mediated by inhibiting drug efflux and reducing virulence. Int J. Antimicrob. Agents. 50, 399–405. 10.1016/j.ijantimicag.2017.03.015 28673609

[B52] Szczepaniak J. Łukaszewicz M. Krasowska A. (2015). Detection of inhibitors of *Candida albicans CDR* transporters using a dis-c3(3) fluorescence. Front. Microbiol. 6:176. 10.3389/fmicb.2015.00176 25806026 PMC4353304

[B53] Tabbene O. Di G. A. Azaiez S. Ben S. I. Elkahoui S. Alfeddy M. N.. (2015). Synergistic fungicidal activity of the lipopeptide bacillomycin d with amphotericin b against pathogenic *Candida* species. FEMS Yeast Res. 15:fov022. 10.1093/femsyr/fov022 25956541

[B54] Tsai M. H. Wang S. H. Hsu J. F. Lin L. C. Chu S. M. Huang H. R.. (2015). Clinical and molecular characteristics of bloodstream infections caused by *Candida albicans* in children from 2003 to 2011. Clin. Microbiol. Infect. 21:1018.e1–8. 10.1016/j.cmi.2015.06.024 26148466

[B55] Tsao S. Rahkhoodaee F. Raymond M. (2009). Relative contributions of the *Candida albicans* ABC transporters Cdr1p and Cdr2p to clinical azole resistance. Antimicrob. Agents Chemother. 53, 1344–1352. 10.1128/AAC.00926-08 19223631 PMC2663127

[B56] Uppuluri P. Nett J. Heitman J. Andes D. (2008). Synergistic effect of calcineurin inhibitors and fluconazole against *Candida albicans* biofilms. Antimicrob. Agents Chemother. 52, 1127–1132. 10.1128/AAC.01397-07 18180354 PMC2258509

[B57] Wei J. Zhang H. Li C. Gang L. Liu X. Wei J. (2016). The calcineruin inhibitor cyclosporine a synergistically enhances the susceptibility of *Candida albicans* biofilms to fluconazole by multiple mechanisms. BMC Microbiol. 16. 113. 10.1186/s12866-016-0728-1 27316338 PMC4912705

[B58] Wong J. H. Lau K. M. Wu Y. O. Ling C. Wong C. W. Yew D. T.. (2015). Antifungal mode of action of macrocarpal c extracted from *eucalyptus globulus* labill (lan an) towards the dermatophyte trichophyton mentagrophytes. Chin. Med. 10, 34. 10.1186/s13020-015-0068-3 26594235 PMC4654844

[B59] Yi X. Hua Q. Yan W. Hua Z. Jun S. Jianjiang X. (2017). Requirement for ergosterol in berberine tolerance underlies synergism of fluconazole and berberine against fluconazole-resistant *Candida albicans* isolates. Front. Cell. Infect. Microbiol. 7:491 10.3389/fcimb.2017.00491 29238700 PMC5712545

