# Supplementary material for: Eucalyptal D Enhances the Antifungal Effect of Fluconazole on Fluconazole-Resistant Candida albicans by Competitively Inhibiting Efflux Pump
Source: Front Cell Infect Microbiol. 2019 Jun 20;9:211. doi: 10.3389/fcimb.2019.00211 (PMC6595430; doi:10.3389/fcimb.2019.00211)
Supplement: Supplementary file 1 [file Table_1.docx]

***Supplementary Material***

**Supplementary Table 1.** List of primers used for RT-PCR in this study.

| Primer name | Sequence |
| --- | --- |
| *ACT1-*F*^a^* | TCCAGCTTTCTACGTTTCCATTC |
| *ACT1-*R | TTGGAACAACGTGAGTAACACCA |
| *CDR1*-F | TGAATACCACGGGTTTGATG |
| *CDR1*-R | TCATGTTCATATGGATTGAC |
| *CDR2*-F | AAAAAGGTGGAAGAACGGC |
| *CDR2*-R | TTGGCATGAGATCCTGGTG |
| *MDR1*-F | GCCGATTACAAACCAACTCT |
| *MDR1*-R | ATCATCATCACCATCCCAAG |
| *ERG1-*F | TTAGAATCATGCCAAACC |
| *ERG1-*R | CCAACTGTCATACCACCC |
| *ERG3-*F | GTCTAATGACCCAGTTGT |
| *ERG3-*R | TCTTCTTCTGCCTTTGCA |
| *ERG5-*F | AGATACCGTCCACCAGTC |
| *ERG5-*R | TGCAAAGCAGGATACAAT |
| *ERG6-*F | GCTACCGTTCATGCTCCA |
| *ERG6-*R | CCATCACCGACTTCAATA |
| *ERG7-*F | GCTTGGGCTTTGATAGGG |
| *ERG7-*R | TCCACTCACCAGTCGGTA |
| *ERG10-*F | TGCCTTGGGTCATCCTCT |
| *ERG10-*R | CCGTTACAAACACCAGCA |
| *ERG11-*F | GAATCCCTGAAACCAAT |
| *ERG11-*R | AGCAGCAGTATCCCATC |
| *ERG13-*F | TGGAACACGCTTACGATT |
| *ERG13-*R | CCAGAATGAGAATCAACGGC |

*^a^*Abbreviations: F, forward primer; R, reverse primer.

**Supplementary Table 2.** The data for relative expression of *CDR1*, *CDR2* and *MDR1* in resistant *C. albicans* 24D with treated of ED, FLC and ED + FLC.

|  | control | | | FLC | | |
| --- | --- | --- | --- | --- | --- | --- |
|  | Mean | SD | N | Mean | SD | N |
| *CDR1* | 1 | 0 | 3 | 0.404134 | 0.0410208 | 3 |
| *CDR2* | 1 | 0 | 3 | 0.729334 | 0.2066158 | 3 |
| *MDR1* | 1 | 0 | 3 | 0.6559911 | 0.153093 | 3 |
|  |  |  |  |  |  |  |
|  |  |  |  |  |  |  |
| ED | | | FLC+ED | | |  |
|  | Mean | SD | N | Mean | SD | N |
| *CDR1* | 1.471903 | 0.189815 | 3 | 0.715455 | 0.163223 | 3 |
| *CDR2* | 3.404132 | 0.383903 | 3 | 1.721453 | 0.321948 | 3 |
| *MDR1* | 1.048194 | 0.097469 | 3 | 0.967246 | 0.114691 | 3 |

**Supplementary Table 3**. The raw data of R6G efflux assay.

The R6G fluorescence of supernatant in the absence and presence of ED

| Time(min) | First time | |  | Second time | |  | Third time | |
| --- | --- | --- | --- | --- | --- | --- | --- | --- |
|  | control | ED |  | control | ED |  | control | ED |
| 5 | 235.6943 | 159.9477 |  | 221.2157 | 74.54833 |  | 177.4067 | 85.42723 |
| 10 | 367.0367 | 219.8831 |  | 229.0408 | 128.4472 |  | 344.0404 | 251.9416 |
| 20 | 427.9918 | 275.3842 |  | 364.8904 | 219.7839 |  | 415.9354 | 304.749 |
| 30 | 572.5143 | 276.6658 |  | 430.5759 | 240.4156 |  | 488.193 | 296.3208 |
| 60 | 702.0449 | 266.4858 |  | 727.5676 | 330.0517 |  | 763.2211 | 355.1208 |

Standard curve: F=299.37C+0.4182, F, fluorescence; C, concentration.

The R6G concentration of supernatant in the absence and presence of ED

|  | control | | | ED (32 μg/ml) | | |
| --- | --- | --- | --- | --- | --- | --- |
| Time (min) | Mean (nmol/ml) | SD | N | Mean (nmol/ml) | SD | N |
| 0 | 0.00 | 0.00 | 3 | 0.00 | 0.00 | 3 |
| 5 | 0.70 | 0.10 | 3 | 0.35 | 0.15 | 3 |
| 10 | 1.04 | 0.24 | 3 | 0.66 | 0.21 | 3 |
| 20 | 1.34 | 0.11 | 3 | 0.88 | 0.14 | 3 |
| 30 | 1.65 | 0.23 | 3 | 0.90 | 0.09 | 3 |
| 60 | 2.44 | 0.10 | 3 | 1.05 | 0.15 | 3 |

**Supplementary Table 4**. MIC values, relative expression levels of resistance gene, and *ERG11* sequence analysis of 5 resistant *C. albicans* isolates.

| *C. albicans* isolates | MIC_80_ | | | Genes overexpressed | Mutation in *ERG11* gene |
| --- | --- | --- | --- | --- | --- |
|  | FLC | KCZ | ICZ |  |  |
| 24D | >128 | 1 | >8 | *CDR1,CDR2,MDR1,ERG11* | D116E  F105F  S137S  L220L  E266D  L370L  N490N |
| 28I | >128 | 16 | >8 | *CDR1,ERG11* | D116E  F105F  S137S  L220L  L370L  N490N |
| CA102 | >128 | 4 | >8 | *CDR1,ERG11* | V437I  F105F  S137S  H183H  L220L  V332V  L370L  Y401Y |
| CA901 | >128 | >16 | >8 | *CDR1,CDR2* | D116E  F105F  S137S  V332V  K342K  L370L  N490N |
| CA112869 | >128 | 16 | >8 | *CDR1,CDR2,MDR1* | V332V  S361S  L370L  Y401Y  D428D  N490N |


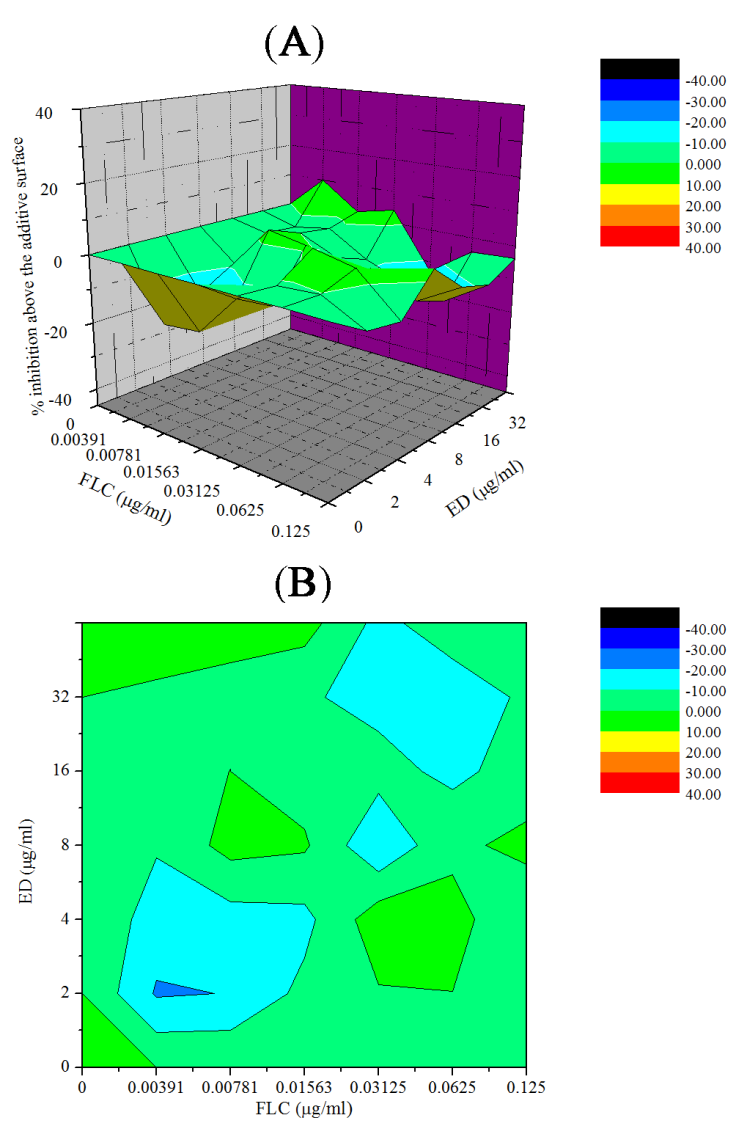


**Supplementary Figure 1.** Interactions of ED and FLC on the double mutant strain DSY659 (*cdr1△/cdr2△*) by checkerboard microdilution assay. The 3D (A) and contour plot (B) was constructed by using OriginPro 7.5. The concentrations of FLC and ED are depicted on the x axis and y axis, respectively, and the percentage growth values obtained for each combination is depicted on the z axis.





**Supplementary Figure 2.** The cytotoxic effect of ED on human normal cell lines by MTT assay. The LO2 cells and MCF 10A cells were treated diﬀerent concentrations of ED. Error bars represent the SD of three experiments.


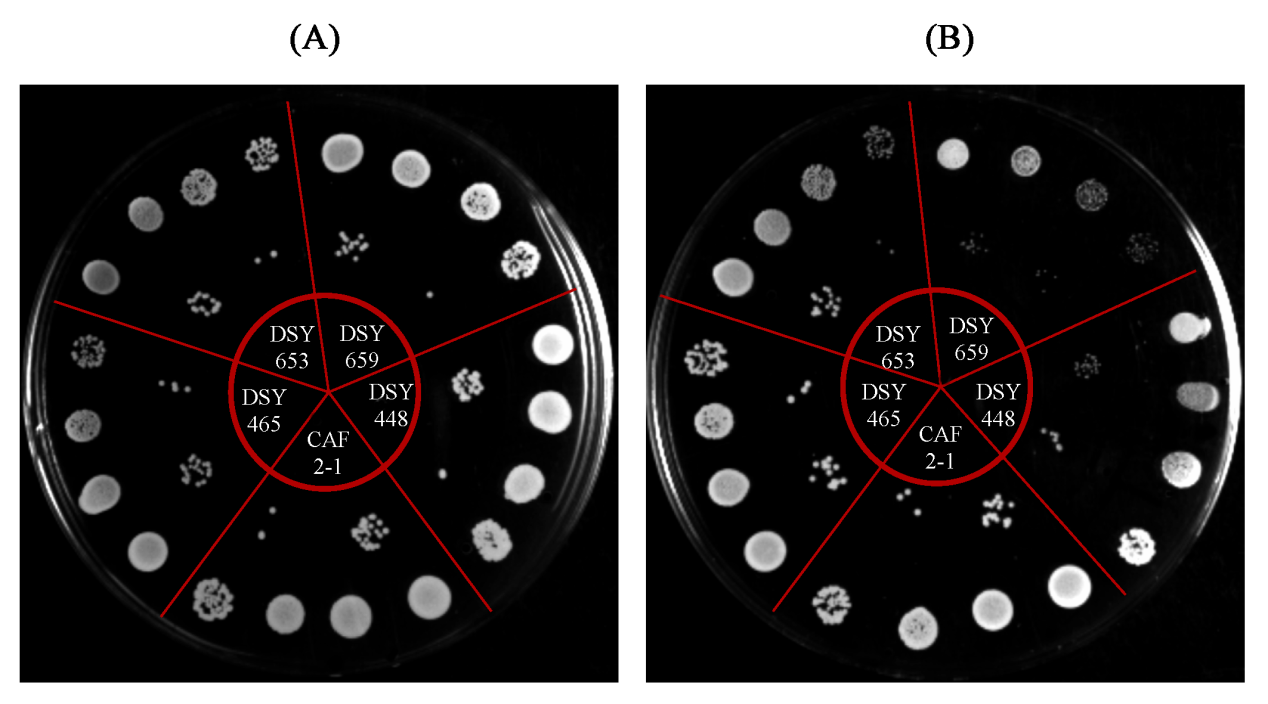


**Supplementary Figure 3.** Original complete picture of the spot assay. The YPD medium containing ED with 1% DMSO (vehicle control) (A) and 128 μg/ml (B).





**Supplementary Figure 4.** Relative expression levels of ergosterol biosynthesis genes *ERG1*, *ERG3*, *ERG5*, *ERG6*, *ERG7*, *ERG10, ERG11* and *ERG13* in resistant *C. albicans* 24D. Cells were treated with FLC (2 μg/ml) and ED (32 μg/ml) alone and combination respectively. Data are means ± SD from three experiments. * *p* <0.05,** *p* ＜0.01, ****p*＜0.001.


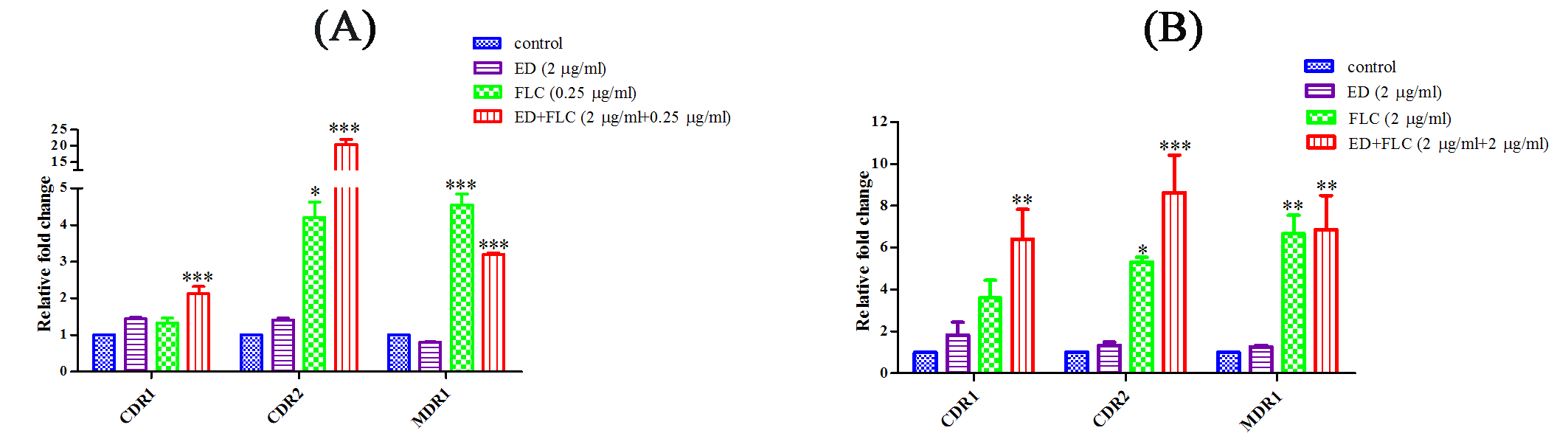


**Supplementary Figure 5.** The relative expression levels of efflux genes *CDR1*, *CDR2*, and *MDR1* on FLC susceptible strain CA21 (A) and FLC resistant strain 28I (B). Cells were treated with FLC, ED and their combination respectively. Data are normalized for control group. *ACT1* was used as an expression control. Relative fold change above 1 represents gene upregulation, while below 1 represents gene downregulation. Data are means ± SD from three experiments. * *p* <0.05，** *p* ＜0.01, ****p*＜0.001.
